# Multidrug-Resistant *Candida haemulonii* and *C. auris*, Tel Aviv, Israel

**DOI:** 10.3201/eid2302.161486

**Published:** 2017-02

**Authors:** Ronen Ben-Ami, Judith Berman, Ana Novikov, Edna Bash, Yael Shachor-Meyouhas, Shiri Zakin, Yasmin Maor, Jalal Tarabia, Vered Schechner, Amos Adler, Talya Finn

**Affiliations:** Tel Aviv University, Tel Aviv, Israel (R. Ben-Ami, J. Berman, S. Zakin, Y. Maor, V. Schechner, A. Adler);; Tel Aviv Sourasky Medical Center, Tel Aviv (R. Ben-Ami, A. Novikov, E. Bash, J. Tarabia, V. Schechner, A. Adler, T. Finn);; Ruth Rappaport Children's Hospital, Haifa, Israel (Y. Shachor-Meyouhas);; Wolfson Medical Center, Holon, Israel (Y. Maor).

**Keywords:** Candida auris, Candida haemulonii, virulence, bloodstream infection, antimicrobial resistance, Israel, fungi

## Abstract

*Candida auris* and *C. haemulonii* are closely related, multidrug-resistant emerging fungal pathogens that are not readily distinguishable with phenotypic assays. We studied *C. auris* and *C. haemulonii* clinical isolates from 2 hospitals in central Israel. *C. auris* was isolated in 5 patients with nosocomial bloodstream infection, and *C. haemulonii* was found as a colonizer of leg wounds at a peripheral vascular disease clinic. Liberal use of topical miconazole and close contact among patients were implicated in *C. haemulonii* transmission. *C. auris* exhibited higher thermotolerance, virulence in a mouse infection model, and ATP-dependent drug efflux activity than *C. haemulonii*. Comparison of ribosomal DNA sequences found that *C. auris* strains from Israel were phylogenetically distinct from isolates from East Asia, South Africa and Kuwait, whereas *C. haemulonii* strains from different countries were closely interrelated. Our findings highlight the pathogenicity of *C. auris* and underscore the need to limit its spread.

## Abstract 2

Clinical features and experimentally deduced virulence indicate that *C. auris* has the greater lethal potential.

*Candida* species are leading causes of bloodstream infection (BSI) in hospitalized patients, particularly those in intensive care units who are exposed to broad-spectrum antimicrobial drugs, indwelling vascular catheters, parenteral nutrition, abdominal surgery, and immunosuppressive agents (*1*,*2*). High rates of attributable death have been associated with delayed initiation of appropriate antifungal treatment (*3*,*4*). This problem is compounded by the emergence of drug-resistant *Candida* species, notably *C. glabrata*, in many hospitals (*5*).

*C. auris* is an emerging opportunistic pathogen, first reported in 2009 as an isolate from the external ear of an inpatient at a hospital in Japan (*6*). It has since been identified as a cause of nosocomial BSI in numerous countries in East Asia, the Middle East, Africa, and Europe (*7*–*11*). *C. auris* might be resistant to multiple classes of antifungal agents and apparently has a potential for person-to-person transmission, challenging clinicians and infection control teams (*12*). *C. auris* often is misidentified by traditional microbiological methods as *C. haemulonii*, a phylogenetically related drug-resistant *Candida* species that also is increasingly reported in healthcare facilities worldwide (*13*).

We report on the detection of multidrug-resistant *C. auris* and *C. haemulonii* in clinical specimens in Tel Aviv, Israel, and specifically on the emergence of *C. auris* as a cause of nosocomial BSI. We highlight distinct clinical and epidemiologic characteristics of these 2 species and present experimental evidence for differences in their virulence.

## Materials and Methods

We undertook this study after *C. auris* BSI was detected in 4 patients during May–October 2014 at the Tel Aviv Sourasky Medical Center (TASMC), a tertiary-level hospital in Tel Aviv. An additional *C. auris* bloodstream isolate was recovered in April 2015 from a patient at the Wolfson Medical Center in Holon (southern Tel Aviv metropolitan area). No additional *C. haemulonii* or *C. auris* isolates were identified through inquiries at additional clinical microbiology laboratories in Israel.

The TASMC Institutional ethics committee approved this study. Need for informed consent was waived because of the observational and anonymous nature of the study.

### Clinical *Candida* isolates

*Candida* isolates recovered from clinical specimens were identified at the TASMC Clinical Microbiology Laboratory by growth characteristics on CHROMagar *Candida* (CHROMagar, Paris, France) and the Vitek 2 YST ID system (bioMérieux, Marcy-l’Étoile, France). The Vitek 2 database does not include *C. auris*, and this species is routinely misidentified as *C. haemulonii* (*13*). We therefore reviewed all isolates identified as *C. haemulonii* during January 2009–August 2015. Isolates recovered during May 2014–August 2015 were stored at −20°C and subjected to further analyses. We assessed thermotolerance by plating serial dilutions of yeast culture on Sabouraud dextrose agar (SDA) plates and assessing growth after 24 h incubation at 35°C–42°C.

### Sequence-Based Species Identification

*Candida* isolates were streaked on SDA plates to ensure purity. We extracted DNA by using PrepMan Ultra solution (Applied Biosystems, Foster City, CA, USA) according to the manufacturer’s instructions and amplified and sequenced the internal transcribed spacer (ITS) and D1/D2 large subunit (LSU) ribosomal DNA segments by using primer pairs ITS1/ITS4 and LSU1/LSU2 (*14*), respectively. PCR was performed in 0.2-mL tubes with 0.4 μmol/L or 0.2 μmol/L of each primer for ITS and LSU, respectively; 10 μL Larova Red Load Taq Master Mix (5×) (Larova, Jena, Germany); and ≈25 ng of template. PCR conditions were 95°C for 4.5 min (denaturation), 40 cycles of 95°C for 30 s (denaturation), 55°C (ITS) or 48°C (LSU) for 30 s (annealing), 72°C for 1 min (extension), and a final extension stage of 72°C for 7 min. PCR products were resolved on 0.7% agarose gel and stained with SERVA DNA stain clear G (Tamar, Mevaseret Zion, Israel). Products were cleaned with QIAquick PCR purification kit (QIAGEN, Hilden, Germany) and sequenced at Hy-Labs (Rehovot, Israel). We then aligned ITS and LSU sequences with the matching type strain sequences for CBS5149^T^ (*C. haemulonii*), CBS7798^T^ (*C. duobushaemulonii*), CNM-CL7239^T^ (*C. haemulonii* var. *vulnera*), CBS10099^T^ (*C. pseudohaemulonii*), and CBS10913^T^ (*C. auris*). A similarity score of >98% in both ITS and LSU sequences was required for species-level identification. All new sequences were deposited in GenBank (Table 1).

**Table 1:** Characteristics of 9 patients with *Candida* isolates available for microbiological analyses, Tel Aviv, Israel*

Isolate ID	GenBank accession nos.		DNA sequence analysis	Patient age, y/sex	Hospital/unit	Date	Comorbidities	Culture source	Clinical significance	Treatment	In-hospital outcome
	ITS	LSU									
TA001-14	KU896955	KU886685	*C. haemulonii*	52/M	TASMC/vascular clinic	2014 Jun	CIDP, steroids, CLU	Wound	Colonization	None	Survival
TA001-15	KU896954	KU886684	*C. haemulonii*	79/M	TASMC/vascular clinic	2015 Apr	PVD, CKD, DMt2, IHD, pacemaker, CLU	Wound	Colonization	None	Survival
TA004-15	KU896956	KU896947	*C. haemulonii*	69/M	TASMC/vascular surgery	2015 Jun	PVD, DMt2, IHD, CLU	Wound	Colonization	None	Survival
TA003-14	KU896949	KU886679	*C. auris*	91/F	TASMC/medicine	2014 May	ANCA vasculitis, M-vent, VAP, steroids, CVC	Blood	CRBSI	Fluconazole	Death
TA002-14	KX518348	KU886682	*C. auris*	79/M	TASMC/neurosurgery	2014 Oct	MDS, ASDH, M-vent, VAP; CVC	Blood	CRBSI	CVC removed; none	Death
TA004-14	KU896950	KU886680	*C. auris*	74/M	TASMC/medicine	2014 Aug	CKD, hemodialysis, CVC, pacemaker, IHD, DMt2	Blood	CRBSI	CVC removed; anidulafungin	Survival
TA005-14	KU896951	KU886681	*C. auris*	57/F	TASMC/medicine	2014 May	PBC	Blood	Primary candidemia	Fluconazole; anidulafungin	Survival
TA003-15	KU896953	KU886683	*C. auris*	42/M	WMC/medicine	2015 Apr	IVDU, HIV, HBV, HCV, MRSA sepsis	Blood	Primary candidemia	Fluconazole; voriconazole	Survival
TA002-15	KU896948	KU886678	*C. auris*	79/M	TASMC/medicine	2015 Apr	CKD, DMt2, IHD, PICC	Urine	Asymptomatic candiduria	None	Survival

*CIDP, chronic inflammatory demyelinating polyneuropathy; CKD, chronic kidney disease; CLU, chronic leg ulcers; CRBSI, catheter-related bloodstream infection; CVC, central venous catheter; DMt2, diabetes mellitus type 2; HBV, hepatitis B virus infection; HCV, hepatitis C virus infection; IHD, ischemic heart disease; ITS, internal transcribed spacer; IVDU, intravenous drug user; LSU, large subunit; MRSA, multidrug-resistant *Staphylococcus aureus*; M-vent, mechanical ventilation; PBC, primary biliary cirrhosis; PICC, peripherally inserted central catheter; PVD, peripheral vascular disease; TASMC, Tel Aviv Sourasky Medical Center; VAP, ventilator-associated pneumonia; WMC, Wolfson Medical Center.

### Phylogenetic Analyses

We aligned ITS and LSU sequences of *C. haemulonii* and *C. auris* isolates by using MUSCLE (*15*) and generated phylogenetic trees with the neighbor-joining method (*16*), using the Kimura 2-parameter method to compute evolutionary distances (*17*). We tested phylogeny with the bootstrap method (500 replicates) and used *Schizosaccharomyces pombe* strains ATCC 38366 and CBS 356 as outgroups. Evolutionary analyses were performed in MEGA7 (*18*).

### Patient Characteristics

We retrospectively reviewed the medical records of patients from whom Vitek-identified *C. haemulonii* was recovered from any site and recorded patient demographics, hospital unit, co-morbidities, medications, and clinical characteristics by using a structured form. We found 40 patient-specific *C. haemulonii* isolates, 20 (50%) of which originated from patients receiving care at an outpatient peripheral vascular disease clinic (clinic A). We therefore conducted an investigation at clinic A, which included review of patient treatment protocols, observed patient care, and surveillance mycologic cultures from environmental surfaces, wound irrigation solutions, dressings, and the hands of medical staff. To define risk factors for *C. haemulonii* colonization, we conducted an unmatched case–control study using 40 noncolonized patients followed at clinic A as controls.

### Antifungal Susceptibility Testing

We determined MICs of fluconazole, itraconazole, voriconazole, posaconazole, amphotericin B, anidulafungin, micafungin, caspofungin, and flucytosine by broth microdilution using Clinical and Laboratory Standards Institute methods (*19*). Results were read after 48 h for azoles, amphotericin B, and flucytosine and after 24 h for echinocandins.

### Rhodamine 6G Efflux

To assess ABC-type drug transporter activity, we determined glucose-induced efflux of rhodamine 6G, as described previously (*20*,*21*). We grew *Candida* isolates to log-phase in liquid yeast extract glucose at 35°C. We then collected yeast cells by centrifugation, transferred 10^9^ cells to 20 mL fresh yeast extract glucose, and incubated them at 27°C for an additional 2 h. Next, we collected yeast cells by centrifugation, washed them twice in phosphate-buffered saline (PBS), and added 10 mL PBS containing 15 μM rhodamine 6G without glucose to the pellets. Suspensions were vortexed and incubated at 27°C for 90 min to enable rhodamine 6G uptake under carbon source–depleted conditions. We then collected cells by centrifugation, washed them twice in PBS, and suspended them in 750 μL PBS in microfuge tubes. To start rhodamine 6G efflux, we added 250 μL PBS with 8 mmol/L glucose. We prepared control tubes with glucose-free PBS, removed them after 5, 15, and 25 min of incubation at 35°C, and measured fluorescence in 200-μL aliquots of supernatant by using a spectrophotometer at excitation 527 nm and emission 555 nm (Synergy HT, BioTek, Winooski, VT, USA).

### Mouse Model of Disseminated Candidiasis

To assess the relative virulence of *C. haemulonii* and *C. auris*, we determined the lethality and tissue fungal loads of representative strains in a mouse model of hematogenous disseminated candidiasis. Experiments were approved by the TASMC Institutional Animal Care and Use Ethics Committee. We used cyclophosphamide (150 mg/kg intraperitoneally) to immunosuppress 6-week-old female BALB/c mice weighing 16–20 g (Harlan, Rehovot, Israel) 3 days before and on the day of infection. *Candida* cells were collected from log-phase culture on the day of infection and washed twice in sterile PBS. Mice were infected in groups of 10 with *C. haemulonii* strain TA001-14, *C. auris* strain TA005-14, and *C. albicans* strain CBS 8837. We injected 100 μL of PBS containing 7 × 10^7^ yeast cells intravenously into the lateral tail vein of each animal. A control group received intravenous injection of cell-free PBS. Death was assessed over 12 days. Kidney tissue fungal loads were determined in separate experiments where mice were similarly immunosuppressed and infected intravenously with 4 × 10^7^ yeast cells/100 μL PBS. Seven days after infection, mice were killed by CO_2_ inhalation, and kidneys were excised aseptically, weighed, and homogenized in a TissueLyser (QIAGEN). Homogenates were serially diluted 10- to 1,000-fold in sterile saline and plated on SDA. We calculated fungal loads (CFU per gram of tissue) from colony counts after 48 h incubation at 35°C.

### Statistical Analyses

We compared continuous variables between case and control patients using the Student *t* test for normally distributed variables and the Wilcoxon rank-sum test for non–normally distributed variables. We compared dichotomous variables using Fisher’s exact test. Rhodamine 6G efflux, expressed as relative fluorescence units, was computed for each *Candida* strain and compared by using 1-way analysis of variance. We used Dunnett’s multiple comparisons test to compare relative fluorescence unit values of specific *C. haemulonii* and *C. auris* strains with averaged control values of 5 *C. glabrata* strains. Survival curves of mice infected with different *Candida* strains were plotted by using the Kaplan-Meier method and compared with the log-rank test. We considered 2-tailed p values <0.05 statistically significant.

## Results

### Sequence-Based Identification

We identified 40 patient-specific *Candida* strains as *C. haemulonii* by the Vitek-2 YST ID system during January 2009–July 2015. Isolates were recovered from wounds (n = 24), urine (n = 9), blood (n = 5), and central venous catheter tips (n = 2). Of these, 9 isolates recovered during May 2014–May 2015 were available for analysis; we identified 6 (including the 5 blood isolates) as *C. auris* and 3 as *C. haemulonii* by ITS and LSU sequencing (Table 1). Sequences were 100% identical among strains of each species. *C. auris* strains from Israel shared 98.6% and 98.3% similarity of ITS and LSU sequences, respectively, with the *C. auris* type strain CBS10913^T^. *C. haemulonii* strains were 100% identical to *C. haemulonii* CBS5149^T^ on the basis of ITS and LSU sequences.

**Figure 1:**
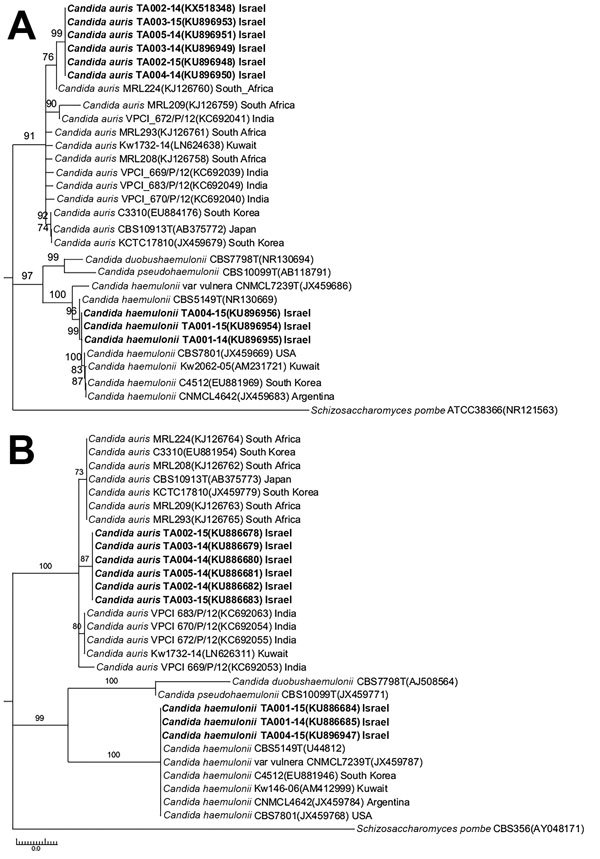
Phylogenetic relationships of *Candida auris* and *C. haemulonii* strains isolated in Tel Aviv, Israel, compared with reference strains. Phylogenetic trees were generated from internal transcribed spacer (A) and D1/D2 domain of the ribosomal DNA large subunit sequences (B). The percentage of replicate trees in which the associated taxa clustered together in the bootstrap test (500 replicates) is shown next to each branch. Bold indicates strains from Tel Aviv. GenBank accession numbers are provided in parentheses. Scale bar indicates nucleotide substitutions per site.

Phylogenetic trees based on ITS and LSU sequences showed that the *C. auris* isolates from Tel Aviv are distinct from other isolates from East Asia, Africa, and the Middle East. Specifically, isolates from Israel showed 98.6% similarity of ITS and LSU sequences with the India clone, represented by CBS12768, 96.2% similarity with the South Korea clone, and 96.7% similarity with strain CH1 from Kuwait. In contrast, ITS and LSU sequences from Israel *C. haemulonii* strains were 100% homologous with *C. haemulonii* from South Korea, Brazil, and Kuwait, suggesting worldwide predominance of a single *C. haemulonii* clone (Figure 1).

### Clinical Features

Eight of 9 patients with sequence-validated isolates were hospitalized at TASMC (Table 1). An additional patient with *C. auris* infection was hospitalized at the Wolfson Medical Center, but was receiving regular care for HIV infection at TASMC. All 3 *C. haemulonii* isolates were recovered from chronic leg ulcers of patients with peripheral vascular disease, 2 of whom were treated at vascular outpatient clinic A. Five of 6 *C. auris* isolates represented BSI: 3 patients had vascular catheter–related candidemia, and 2 had primary nosocomial candidemia of unclear origin. Two of 5 patients with *C. auris* BSI died during hospitalization.

We reviewed the medical records of 40 patients with Vitek-identified *C. haemulonii* cultures. Thirty-three (83%) were male. Median age was 74 years (range 37–91 years). Nineteen (48%) had peripheral vascular disease, 20 (50%) had diabetes mellitus, 22 (55%) had ischemic heart disease, and 11 (28%) had end-stage renal disease. Twenty patients (50%) were receiving regular care at clinic A, representing 8% (20/261) of all clinic patients. In all 20 patients, *C. haemulonii* had been recovered from chronic leg ulcers, and none had documented wound infection at the time of culture. Cultures of environmental surfaces, medical devices, dressings, irrigation solutions, and hands of medical staff were negative for yeast. Compared with 40 control patients who were not carriers of *C. haemulonii*, carriers were older, had a lower glomerular filtration rate, and were more likely to be male and to have ischemic heart disease (Table 2). Observations revealed a practice among medical staff of routinely applying topical miconazole cream to chronic ulcers without evidence of infection. Periodic wound cultures were obtained regularly, irrespective of signs of ulcer inflammation or purulence. Multiple social interactions were noted among patients in a single room where wound care was performed.

**Table 2:** Comparison of colonized and noncolonized patients with *Candida haemulonii*, clinic A, Tel Aviv Sourasky Medical Center, Tel Aviv, Israel*

p value	Odds ratio (95% CI)	Controls, n = 40	Cases, n = 20	Variable
0.015	NA	63.0 (43–94)	77.5 (44–91)	Median age, y (range)
0.017	6.65 (1.26–65.0)	23 (57.5)	18 (90)	Male sex
0.44	NA	48 (9–192)	40 (8–228)	Median time in clinic A, mo (range)
0.022	NA	62.9 ± 3.61	47.7 ± 5.56	eGFR, mL/min/1.73m^2^, mean ± SEM
0.057	3.05 (0.88–11.2)	15 (37.5)	13 (65)	Chronic kidney disease, stage 3–4
0.069	3.85 (0.76–21.0)	4 (10)	6 (30)	Dialysis
0.003	5.5 (1.51–21.1)	10 (25)	13 (65)	Ischemic heart disease
0.27	2.05 (0.59–7.37)	19 (47.5)	13 (65)	Diabetes mellitus
1.00	0.80 (0.13–5.84)	35 (87.5)	17 (85)	Peripheral vascular disease

*Values are no. (%) patients except as indicated. eGFR, glomerular filtration rate, estimated using the Modification of Diet in Renal Disease (MDRD) equation (*22*); NA, odds ratio is not applicable for continuous variables.

### Antifungal Susceptibility

All *C. haemulonii* and *C. auris* isolates had fluconazole MICs >8 mg/L (range 16–64 mg/L; MIC_50_ 32 mg/L). MICs of other azoles were also elevated: itraconazole, 0.25 to >16 mg/L (MIC_50_ 0.5 mg/L); voriconazole, 0.25–1 mg/L (MIC_50_ 0.5 mg/L); and posaconazole, 0.06 to >8 mg/L (MIC_50_ 0.25 mg/L). Amphotericin B MIC ranged from 1 to 2 mg/L for *C. auris* isolates and from 2 to 8 mg/L for *C. haemulonii* isolates. All isolates appeared susceptible to anidulafungin (MIC 0.03 mg/L) and all isolates except 1 *C. haemulonii* were susceptible to micafungin (MIC 0.12–0.5 mg/L; MIC_50_ 0.12 mg/L). Caspofungin MIC was 0.5 mg/L for all isolates. All isolates except 1 *C. auris* were susceptible to flucytosine (Table 3).

**Table 3:** Antifungal susceptibility profiles of *Candida haemulonii* and *C. auris* isolates, Tel Aviv, Israel*

Isolate ID	Species	MIC, mg/L								
FLZ	ITZ	VRZ	PSZ	AMB	ANF	MCF	CSF	FC
TA001–14	*C. haemulonii*	16	>16	0.25	>8	2	0.03	0.5	0.5	0.12
TA001–15	*C. haemulonii*	64	1	0.5	0.5	8	0.03	0.12	0.5	0.12
TA004–15	*C. haemulonii*	16	0.25	1	0.06	8	0.03	0.12	0.5	0.12
TA003–14	*C. auris*	32	0.5	0.5	0.5	1	0.03	0.12	0.5	0.5
TA002–14	*C. auris*	64	0.5	0.5	0.5	2	0.03	0.12	0.5	0.25
TA004–14	*C. auris*	64	0.5	0.5	0.25	2	0.03	0.12	0.5	0.25
TA005–14	*C. auris*	32	0.5	0.5	0.25	2	0.03	0.12	0.5	0.5
TA002–15	*C. auris*	32	0.5	0.5	0.12	2	0.03	0.25	0.5	1
TA003–15	*C. auris*	64	0.5	1	0.12	2	0.03	0.12	0.5	0.5

*AMB, amphotericin B; ANF, anidulafungin; CSF, caspofungin; FC, flucytosine; FLZ, fluconazole; ITZ, itraconazole; MCF, micafungin; PSZ, posaconazole; VRZ, voriconazole.

### Rhodamine 6G Efflux

**Figure 2:**
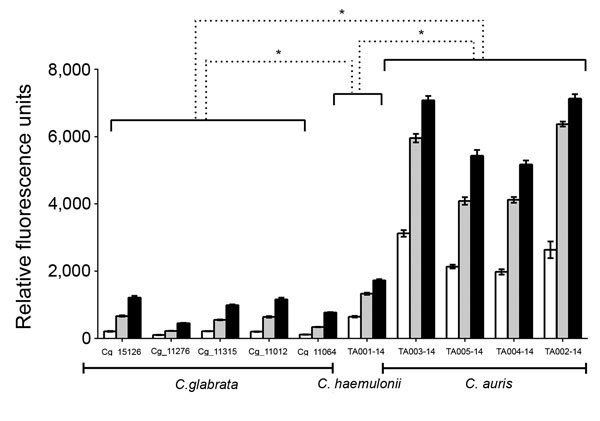
Comparison of rhodamine 6G efflux over time among *Candida* isolates from Tel Aviv, Israel. Rhodamine 6G efflux is expressed as relative fluorescence units measured in culture supernatants after the addition of 8 mM glucose. Statistical significance was measured with 1-way analysis of variance and Dunnett’s post-test comparing each *C. haemulonii* and *C. auris* strain with the averaged value of *C. glabrata* strains at the corresponding time point. White bars, 5 min; gray bars, 15 min; black bars, 25 min. *p<0.0001.

Rhodamine 6G is a substrate of ATP binding cassette (ABC) type efflux pumps responsible for multiazole resistance in *C. glabrata*. *C. haemulonii* and *C. auris* strains exhibited robust rhodamine 6G efflux activity when glucose (8 mM) was present in the medium, consistent with ABC-type transport. Rhodamine 6G efflux of *C. auris* strains was significantly greater than that of *C. glabrata* strains (14.4-, 10-, and 6.7-fold higher at 5, 15, and 25 min, respectively; p<0.0001) and *C. haemulonii* (3.8-, 3.8-, and 3.6-fold higher at 5, 15, and 25 min, respectively; p<0.0001). *C. haemulonii* showed greater rhodamine 6G efflux than *C. glabrata* (3.8-, 2.7-, and 1.9-fold higher at 5, 15, and 25 min, respectively (p<0.0001) (Figure 2).

### Thermotolerance

**Figure 3:**
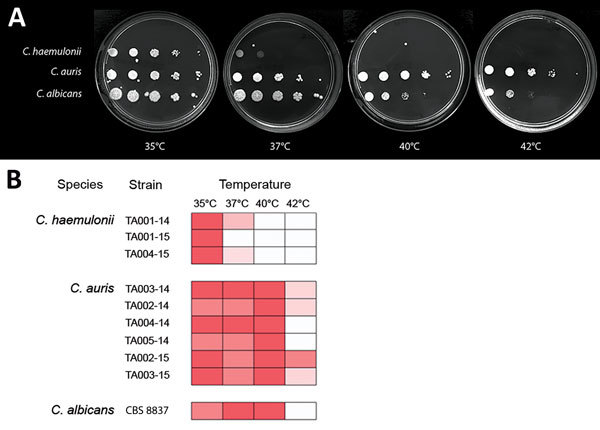
Differing thermotolerance of *Candida auris* and *C. haemulonii*. A) Sabouraud dextrose agar plates showing growth of representative *Candida* strains after 24 h incubation at 35°C–42°C; B) Thermal growth range of *Candida* isolates from Tel Aviv, Israel.

Survival and growth at physiologic temperature are prerequisites for microbial invasion and pathogenicity. *C. haemulonii* isolates grew well at 35°C, but growth at 37°C was poor or absent, and no growth occurred at 40°C and 42°C. In contrast, growth of *C. auris* isolates at 37°C and 40°C was similar to that of *C. albicans*, and 4 of 6 isolates grew at 42°C (Figure 3).

### Virulence in a Mouse Model of Disseminated Candidiasis

**Figure 4:**
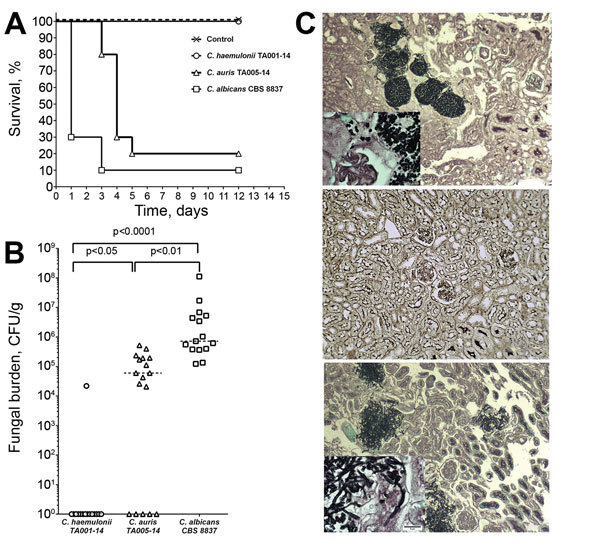
Differing virulence of *Candida auris* and *C. haemulonii* assessed in a mouse model of hematogenous disseminated candidiasis. Virulence was assessed in immunosuppressed BALB/c mice after intravenous injection of yeast cell suspension. A) Survival curves showing significantly shorter survival of mice infected with *C. albicans* than *C. auris* and no death among mice infected with *C. haemulonii*. B) Kidney fungal load (CFU per gram of tissue) shown to be significantly higher in mice infected with *C. albicans* than in those infected with *C. auris*, whereas no viable yeast was cultured from kidneys of mice infected with *C. haemulonii*. C) In mouse kidneys, *C. auris* cells formed aggregates and no hyphae (top) whereas *C. albicans* formed extensive tissue-invasive hyphae (bottom); *C. haemulonii* was not detected in tissue sections (middle). Grocott methenamine silver staining, original magnification ×100 for panels, ×400 for insets.

We compared the virulence of *C. auris* and *C. haemulonii* isolates in a mouse model of hematogenous disseminated candidiasis. *C. haemulonii* was completely nonvirulent in this model; 100% of mice survived 12 days after inoculation with no visible signs of illness. In contrast, inoculation with *C. auris* resulted in rapid death and only 20% survival 5 days after infection (p = 0.0002, log-rank test). Death of mice infected with *C. auris* was significantly less rapid than that of mice infected with *C. albicans* (median survival 4 d and 1 d, respectively; p = 0.01; Figure 4, panel A). Kidney tissue fungal load correlated with survival rates. Specifically, we recovered no viable yeast cells from kidneys of mice inoculated with *C. haemulonii*, whereas infection with *C. auris* and *C. albicans* yielded median tissue loads of 5.9 × 10^4^ CFU/g and 7.1 × 10^5^ CFU/g, respectively (p<0.0001; Figure 4, panel B). Histopathologic analysis showed yeast cell aggregates in kidneys of *C. auris*–inoculated mice, distinct from tissue invasive hyphae observed in *C. albicans*–infected kidneys (Figure 4, panel C).

## Discussion

Concern about the international emergence and spread of *C. auris* as a cause of invasive infection in hospitals stems from 3 characteristics of this opportunistic pathogen (*12*,*13*): 1) resistance to multiple antifungal drugs and possibly to all major classes of systemic antifungal drugs; 2) horizontal transmission among hospitalized patients, leading to nosocomial outbreaks (*8*,*10*,*11*,*13*); and 3) high associated death rates (*7*,*8*,*10*). *C. auris* and *C. haemulonii* are phylogenetically related species in the Metschnikowia clade that share a propensity for multidrug resistance. We identified *C. auris* and *C. haemulonii* in 2 hospitals in Israel and highlighted clinical and experimental evidence for differences in the drug-susceptibility patterns, drug efflux activity, pathogenicity, and global phylogenetics of these 2 species.

In our study, *C. auris* and *C. haemulonii* had high MICs of azoles and amphotericin B. Echinocandin MICs were within the susceptible range. An amphotericin B epidemiologic cutoff value of 2 mg/L previously was established (*23*), but clinical correlation between amphotericin B MIC and treatment outcomes is lacking (*24*). Compared with *C. auris*, *C. haemulonii* isolates had higher amphotericin B MICs. The relevance of these resistance patterns to treatment strategies remains to be determined.

ABC-type efflux activity, as evidenced by Rhodamine 6G transport, was significantly greater among *C. auris* than *C. glabrata* isolates. This observation provides a mechanistic basis for the intrinsic resistance of *C. auris* to azoles and is consistent with the identification of multiple putative transporter-encoding genes belonging to the ABC and major facilitator gene families in the *C. auris* genome (*25*).

Of Vitek-identified *C. haemulonii* isolates at TASMC, 50% were wound cultures from patients cared for at clinic A. That most of these isolates were not available for sequencing is a limitation of our study. However, we identified the 2 *Candida* isolates from clinic A patients that were available by ITS and LSU sequencing as *C. haemulonii*, and all 3 sequence-identified *C. haemulonii* isolates were recovered from leg ulcers of patients with peripheral vascular disease. Colonization of patients treated in close proximity in 1 room strongly suggests person-to-person transmission and supports interim guidelines for contact isolation (*26*). However, we were unable to identify an environmental reservoir of *C. haemulonii*. We suggest that topical application of miconazole to wounds most likely caused selective pressure and facilitated the overgrowth of *C. haemulonii*. After this investigation and termination of routine topical azole use, no additional cases of *C. haemulonii* were detected in clinic A during April 2015–July 2016.

We recovered 5 of 6 sequence-identified *C. auris* isolates from patients with nosocomial BSI. In contrast, all *C. haemulonii* isolates were cultured from superficial wounds. This observation reflects the global epidemiology of these species. *C. haemulonii* has been isolated from chronic leg ulcers of patients in India and Brazil (*27*,*28*). *C. auris* has caused outbreaks of BSI in the United Kingdom (*13*), India (*8*), Kenya (*11*), South Africa (*9*), and South Korea (*29*), whereas reports of *C. haemulonii* as an agent of BSI have been infrequent (*27*,*29*–*32*). Moreover, *C. auris* fungemia is associated with high death rates (*8*,*10*), contrasting with reports of patients surviving prolonged *C. haemulonii* fungemia (*31*). Fatal *C. haemulonii* fungemia, although rare, has been reported in neonates and in patients with cancer and neutropenia (*27*,*32*).

In our study, *C. auris*, but not *C. haemulonii*, grew at 37°C–42°C and exhibited lethality and tissue invasion in a mouse model of invasive candidiasis only slightly less than those of *C. albicans*, the prototypical pathogenic *Candida* species. Both *C. auris* and *C. haemulonii* are unable to form hyphae, which contribute to virulence in *C. albicans*. Formation of large aggregates resulting from failure of budding yeast to separate has been noted in some *C. auris* isolates (*33*). We observed distinct yeast cell aggregates in the kidneys of mice with lethal *C. auris* infection, which suggests that aggregation might be a mode of immune evasion and persistence in tissue. The *C. auris* genome contains *C. albicans* gene orthologs, such as secreted proteinases and mannosyl transferases, which might have roles in pathogenesis (*25*). However, *C. auris* has a genome that is highly divergent from those of other *Candida* species, and most of its genes have not yet been characterized (*25*).

Ribosomal DNA sequences were identical among *C. haemulonii* strains from Israel, Kuwait, East Asia, South America, and the United States. In contrast, the global phylogenetics of *C. auris* demonstrate distinct clones for each country, indicating greater genomic diversity for this species. Further study is needed to establish whether the divergence of *C. auris* clones translates into country-specific patterns of invasiveness, virulence, and drug resistance. Our findings affirm the need for intensified vigilance and mobilization of infection control measures to limit the spread of *C. auris*.
